# Co-Design of a Voice-Based Digital Health Solution to Monitor Persisting Symptoms Related to COVID-19 (UpcomingVoice Study): Protocol for a Mixed Methods Study

**DOI:** 10.2196/46103

**Published:** 2023-06-19

**Authors:** Aurelie Fischer, Gloria A Aguayo, Pauline Oustric, Laurent Morin, Jerome Larche, Charles Benoy, Guy Fagherazzi

**Affiliations:** 1 Deep Digital Phenotyping Research Unit Department of Precision Health Luxembourg Institute of Health Strassen Luxembourg; 2 Université de Lorraine Nancy France; 3 Association ApresJ20 COVID Long France Luce France; 4 Fédération des Acteurs de la Coordination en Santé-Occitanie, Hôpital La Grave Toulouse France; 5 Centre Hospitalier Neuro-Psychiatrique Ettelbruck Luxembourg; 6 Universitäre Psychiatrische Kliniken Basel Basel Switzerland

**Keywords:** COVID-19, long COVID symptoms, vocal biomarkers, digital health, co-design, mixed methods, mobile phone

## Abstract

**Background:**

Between 10% and 20% of people with a COVID-19 infection will develop the so-called *long COVID syndrome*, which is characterized by fluctuating symptoms. Long COVID has a high impact on the quality of life of affected people, who often feel abandoned by the health care system and are demanding new tools to help them manage their symptoms. New digital monitoring solutions could allow them to visualize the evolution of their symptoms and could be tools to communicate with health care professionals (HCPs). The use of voice and vocal biomarkers could facilitate the accurate and objective monitoring of persisting and fluctuating symptoms. However, to assess the needs and ensure acceptance of this innovative approach by its potential users—people with persisting COVID-19–related symptoms, with or without a long COVID diagnosis, and HCPs involved in long COVID care—it is crucial to include them in the entire development process.

**Objective:**

In the UpcomingVoice study, we aimed to define the most relevant aspects of daily life that people with long COVID would like to be improved, assess how the use of voice and vocal biomarkers could be a potential solution to help them, and determine the general specifications and specific items of a digital health solution to monitor long COVID symptoms using vocal biomarkers with its end users.

**Methods:**

UpcomingVoice is a cross-sectional mixed methods study and consists of a quantitative web-based survey followed by a qualitative phase based on semistructured individual interviews and focus groups. People with long COVID and HCPs in charge of patients with long COVID will be invited to participate in this fully web-based study. The quantitative data collected from the survey will be analyzed using descriptive statistics. Qualitative data from the individual interviews and the focus groups will be transcribed and analyzed using a thematic analysis approach.

**Results:**

The study was approved by the National Research Ethics Committee of Luxembourg (number 202208/04) in August 2022 and started in October 2022 with the launch of the web-based survey. Data collection will be completed in September 2023, and the results will be published in 2024.

**Conclusions:**

This mixed methods study will identify the needs of people affected by long COVID in their daily lives and describe the main symptoms or problems that would need to be monitored and improved. We will determine how using voice and vocal biomarkers could meet these needs and codevelop a tailored voice-based digital health solution with its future end users. This project will contribute to improving the quality of life and care of people with long COVID. The potential transferability to other diseases will be explored, which will contribute to the deployment of vocal biomarkers in general.

**Trial Registration:**

ClinicalTrials.gov NCT05546918; https://clinicaltrials.gov/ct2/show/NCT05546918

**International Registered Report Identifier (IRRID):**

DERR1-10.2196/46103

## Introduction

### Context

#### COVID-19 and Long COVID

The novel SARS-CoV-2 infected >532 million people and caused >6.3 million deaths worldwide as of June 2022 [1].

COVID-19 initial infection can take several forms, from asymptomatic to moderate or severe illness. Common symptoms are fever, cough, dyspnea, fatigue, loss of taste or smell, and gastrointestinal symptoms. Complications include acute respiratory distress syndrome, anemia, or acute cardiac injury [2].

After the acute phase, some people with COVID-19 develop a so-called *long COVID* or *post-COVID condition*. The World Health Organization’s definition of this condition is as follows: “Post-COVID-19 condition occurs in individuals with a history of probable or confirmed SARS-CoV-2 infection, usually 3 months from the onset of COVID-19 with symptoms that last for at least 2 months and cannot be explained by an alternative diagnosis. Common symptoms include fatigue, shortness of breath, and cognitive dysfunction but also others that generally have an impact on everyday functioning. Symptoms may be new onset, following initial recovery from an acute COVID-19 episode, or persist from the initial illness. Symptoms may also fluctuate or relapse over time” [3,4].

It has been estimated that a mean of 10% to 20% of patients with COVID-19, representing millions of people worldwide, will have persisting or relapsing symptoms >12 weeks after acute infection [5] with complaints such as tachycardia, extreme fatigue, or dyspnea as observed by Rubin [6]. In Luxembourg, we showed that 59% of COVID-19–infected people included in the Predi-COVID cohort study, who completed a detailed 1-year questionnaire, still declared 1 or more persisting symptoms. The number of persisting symptoms increased with the initial disease severity, and the quality of life of these participants was highly affected by sleep disorders (54%) and altered respiratory quality of life (12.9%) [7].

The COVID-19 pandemic accelerated the use of remote patient monitoring in clinical practice or research for safety and emergency reasons, justifying the need for innovative digital health solutions to monitor key parameters or symptoms related to COVID-19 or long COVID. A panel of experts from the National Institute for Health and Care Excellence recommended the development of telemonitoring and encouraged self-management of acute and long COVID symptoms in a tailored and accessible way for each patient [8].

Although long COVID is now a recognized illness in many countries, few dedicated consultations exist, and the awareness and scientific knowledge of this disease are still poor among not only the general population but also general practitioners. For these reasons, many people experiencing persisting symptoms after a COVID-19 infection (confirmed or not) may feel abandoned by or lost in the health care system, with difficulties in obtaining a long COVID diagnosis or obtaining specific support, and there is a demand among them for new monitoring solutions to objectify the symptom evolution and identify causes of symptom degradation. Moreover, people with long COVID experience difficulties in performing daily physical tasks, and many of them cannot engage in the same levels of activity or work as before [9].

#### Vocal Biomarkers of COVID-19 Symptoms

Using voice is an interesting approach for telemonitoring, as it is easy to collect, quick, and energy efficient, inducing less fatigue on the patients. Moreover, the use of voice-based technologies is increasing swiftly; in 2019, 31% of smartphone users worldwide used voice technology at least once a week, and 20% of queries on Google’s mobile app and Android devices were voice searches [10].

Voice is a rich source of health-related information, as many voice features can be related to symptoms or health status [11-14]. In addition, because voice analysis involves highly complex methods for processing audio features, this kind of development might also be capable of detecting subtle changes associated with COVID-19 symptoms [10].

For example, voice features have previously been associated with a COVID-19 infection or a consequence of COVID-19 infection [15,16]. COVID-19 infection or complications can affect the voice through different mechanisms. Indeed, respiratory insufficiency can lead to reduced airflow and thus to changes in voice parameters. Other studies showed that voice quality was reduced in patients with COVID-19 owing to repeated cough, laryngeal or pharyngeal erythema, or sore throat [17-19].

In Luxembourg, participants from the Predi-COVID hybrid prospective cohort study were invited to perform voice recordings at the same time as they completed web-based questionnaires regarding their symptoms and health status. To date, almost 6000 voice recordings from more than >500 patients with COVID-19 have already been collected in the Predi-COVID study [2]. These voice recordings have been analyzed, and promising vocal biomarker candidates for fatigue, loss of taste and smell, and symptomatic status in people with a COVID-19 infection have already been identified [20-22].

The question now is to determine how these vocal biomarkers could help and improve the quality of life of people with long COVID and how they could be implemented in a digital health solution such as a smartphone app. For example, people with COVID-19 or long COVID symptoms could record their voice in real-life settings by following basic instructions such as “read a pre-defined text, count from 1 to 20, or say a vowel as long as possible,” and the app would analyze voice features and give them back a result for one or several vocal biomarkers. Importantly, to ensure that the developed digital solution using vocal biomarkers is meaningful and useful for the patients, our study involves them at an early stage together with health care professionals (HCPs).

### Objectives

The UpcomingVoice project aims to co-design a voice-based digital health solution for screening and self-monitoring of frequently reported COVID-19–related symptoms with its end users, namely, (1) people with persisting COVID-19–related symptoms, with or without a long COVID diagnosis confirmed by an HCP, and (2) HCPs involved in the care of people with long COVID. The goal of this study is to develop a digital health solution that really matters and helps people with long COVID in their daily lives and could be a complementary support in addition to the medical care that they could benefit from.

To achieve this main objective, the UpcomingVoice study will (1) explore the impact of long COVID in the everyday lives of people with long COVID and the needs of people with long COVID to improve their quality of life and manage their symptoms; (2) explore the extent to which the use of voice and, in particular, vocal biomarkers could propose a solution to the patient’s needs (ie, we will investigate the expectancies, acceptability, fears, barriers, and leverages regarding using voice to self-monitor or screen for long COVID symptoms); and (3) define the specifications that such a mobile app should meet so that it could be recommended to patients by HCPs and be considered acceptable and useful by its intended users in terms of technological aspects (ie, type of device, type of voice recordings, etc), frequency of use, design, etc.

As a secondary aim, we will also assess the transferability potential of the results of this study in the context of COVID-19 to other diseases (eg, monitoring fatigue could also be used in the context of cancer).

## Methods

### Study Design

**Figure 1:**
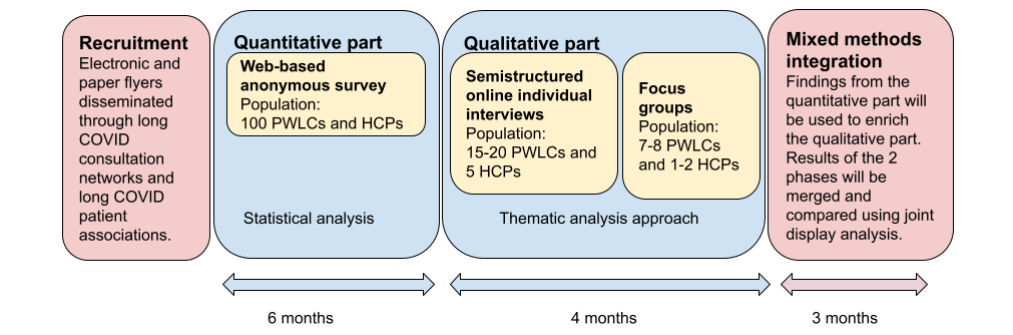
Study design. HCP: health care professional; PWLC: people with long COVID.

This cross-sectional study is based on a mixed method sequential explanatory design. It consists of 2 successive phases: a quantitative phase (descriptive approach), followed by a qualitative phase (inductive pragmatic approach). The study design is summarized in Figure 1. The research questions addressed in the 2 phases of the study are listed in Table 1.

A conceptual framework of the key areas of daily functioning will be elaborated based on the literature review, the survey, and individual interview results as previously described [23], and the framework will be used to identify the most relevant use cases of the digital health solution. The general design of the digital health solution will be defined using the survey results, and its specific items and response modalities will be further elaborated during the qualitative phase.

To study the transferability potential of the digital health solution that will be developed, we will provide an extensive description of our study population and their daily life concerns to identify other populations, diseases, or conditions with similar characteristics (in terms of impact on quality of life, main symptoms, and intensity of symptoms) that could also benefit from a voice-based technology. Subsequently, we will use the APEASE (Affordability, Practicability, Effectiveness, Acceptability, Side Effects, and Equity) criteria and other relevant checklists previously described for the evaluation of transferability of health technology assessments [24,25] to assess the potential transferability of each use case or the main characteristics of the solution identified in the 2 phases of the study to the other fields.

**Table 1:** Research questions (RQs) addressed in phase 1 and phase 2.

Study phase and objectives			Measure instrument		RQs		Questions of HCP^a^ survey		Questions of PWLC^b^ survey
**Phase 1 (quantitative phase)**									
	To understand the needs of PWLC and to assess acceptability, expectancies, and fears regarding the use of voice to meet these needs	Web-based anonymous survey (different questions for PWLC and HCPs)		1. What are the demographic and sociodemographic characteristics of the participants?		1-3		1-3	
	To define the general specifications of the digital health solution based on vocal biomarkers	Web-based anonymous survey (different questions for PWLC and HCPs)		2. What is the acceptance rate of the use of voice for symptom monitoring? 3. What proportion of participants already heard about the notion of vocal biomarker? 4. What are the most important LC^c^ symptoms to be monitored? 5. What are the needs, fears, expectations, and use cases for PWLC and HCPs regarding the use of voice for symptom monitoring? 6. What are the main specifications of a smartphone app based on VB^d^ (engagement, functionalities, esthetics, information content, and subjective items) 7. What are the most important aspects of the smartphone app? 8. Should such an app be reimbursed or be paid by the patient? What cost would be acceptable? 9. To what extent do COVID-19 infection and vaccination have an impact on the opinions and needs regarding the use of voice for symptom monitoring?		5^e^ 4^f^ 11-12^g^ 6-10; 14^h^ 19-35^i^ 13^j^ 15-18^k^		17^e^ 16^f^ 24-25^g^ 18-27^h^ 32-57^i^ 26^j^ 28-31^k^ 4-15^l^	
**Phase 2 (qualitative phase)**									
	To further clarify PWLC's needs and their expectations and fears regarding voice-based technologies	Individual semistructured interviews with PWLC and HCPs		10. What are the needs and problems of PWLC in their everyday lives and how could the use of vocal biomarkers bring a solution to these problems?		N/A^m^		N/A	
	To define the specifications of the voice-based digital health solution	Individual semistructured interviews with PWLC and HCPs		11. What are the detailed specifications of a smartphone app based on VB (engagement, functionalities, esthetics, information content, and subjective items)? 12. What are the best app items and response options?		N/A		N/A	
	To develop a smartphone app prototype	2 focus groups with a panel of 8-10 PWLC and HCPs together		13. How is the smartphone app prototype perceived? 14. What are the improvements suggested by PWLC and HCPs?		N/A		N/A	

^a^HCP: health care professional.

^b^PWLC: people with long COVID.

^c^LC: long COVID.

^d^VB: vocal biomarkers.

^e^Survey questions related to RQ2.

^f^Survey questions related to RQ3.

^g^Survey questions related to RQ4.

^h^Survey questions related to RQ5.

^i^Survey questions related to RQ6.

^j^Survey questions related to RQ7.

^k^Survey questions related to RQ8.

^l^Survey questions related to RQ9.

^m^N/A: not applicable.

### Phase 1 (Quantitative)

The first phase, the quantitative phase, consists of a web-based anonymous survey.

The objective of this survey is to define the main aspects of daily life impacted by long COVID and the needs of people with long COVID and to assess the acceptability and expectancy toward the use of vocal biomarkers for the symptom monitoring of long COVID. The general outline of the digital health solution based on vocal biomarkers will be defined based on these results.

The following dimensions adapted from the user version of the Mobile Application Rating Scale (uMARS) [26] will be covered by different questions: engagement, functionality, aesthetics, information, and subjective items (“Would you recommend,” “Would you be interested in,” etc).

### People With Long COVID

In addition to the above-mentioned items, the survey for people with long COVID has been designed to further identify the most impairing symptoms for their quality of life, to assess whether the severity of their initial infection or of their long COVID symptoms or their vaccination status has an impact on their opinions and needs regarding a new health technology based on voice, and to assess how the use of vocal biomarkers could be of interest for the management of their symptoms. The detailed questionnaire for people with long COVID is provided in Multimedia Appendix 1.

### HCP Survey

The HCP survey is focused on assessing the needs of their patients with long COVID in their daily lives and how they foresee the use of vocal biomarkers of long COVID symptoms in the management of their patients and in the health care system. The detailed HCP questionnaire is provided in Multimedia Appendix 2. Completing the survey will take a maximum of 20 minutes to ensure a high acceptability rate, particularly in people with long COVID who frequently experience fatigue and trouble concentrating. The participants are expected to complete the survey once.

Web-based survey data will be collected and managed using REDCap (Research Electronic Data Capture; Vanderbilt University) tools hosted at the Luxembourg Institute of Health (LIH) [27].

### Phase 2 (Qualitative)

The second phase is based on qualitative methods and consists of semistructured individual interviews and focus groups. Participants in this phase will have either participated in the web-based survey or not.

Individual interviews will be conducted to deeply understand the impact of long COVID on daily life, the needs of people with long COVID, and the potential interest in using vocal biomarkers to meet these needs, and define use cases for the digital health solution. The maximum duration for the interview will be 60 minutes.

Following the individual interviews, 2 successive focus groups (maximum duration of 90 minutes) will be organized with a panel comprising people with long COVID and HCPs. During the first focus group, the objective is to present 1 or 2 prototypes of the digital health solution and to collect users’ comments and suggestions. The prototype or prototypes will be improved according to the collected comments and validated during the interaction with the second focus group.

The individual interview and focus group guides will be elaborated based on the insights obtained from the survey in phase 1. The questions will be asked in a general manner to ensure the identification of the most relevant aspects for people with long COVID in their daily lives and to minimize the expectancy and confirmatory bias.

Using teleconferencing software, interviews and focus groups will be conducted by a qualified interviewer and a moderator, respectively, both trained in qualitative methods.

### Patient Public Involvement

The INVOLVE recommendations [28] will be followed to involve people with long COVID and HCPs in the most efficient way during the entire course of the project. First, we contacted a representative of a long COVID association and an HCP in charge of people with long COVID and presented them with the study aims and the participation procedures. They were then invited to review the study protocol, participant documents, and questionnaires before the study implementation.

Second, we will also follow the framework of core principles for selecting and developing measurements in digital health developed by Manta et al [29]. The 4 levels of this framework to evaluate the meaningfulness of digital biomarkers for patients are the Meaningful Aspect of Health, the Concept of Interest, the Outcome to be measured, and the End point.

In this perspective, the UpcomingVoice study design is a co-design process that will continuously involve patients with long COVID and HCPs.

At each step, the participants will receive feedback on the results from the previous step: during individual interviews, feedback on survey results will be given, and during the first focus group, results from the individual interviews will be provided and discussed.

Finally, participants with long COVID and HCPs willing to be even more deeply involved in our research will be invited to coauthor the scientific articles presenting the results of this research and to promote the results of the study to the lay audience.

### Study Participants

#### Inclusion Criteria

Participation in the study will be proposed to adults (men and women) with persisting COVID-19–related symptoms, with or without a long COVID diagnosis, and to HCPs in charge of patients with long COVID (doctors, nurses, psychologists, physiotherapists, etc).

All participants must be aged >18 years, have a sufficient level of speaking and comprehension in French, and have internet access to participate in the web-based survey and the web-based individual interviews and focus groups.

Seeking a representative sample of people is not part of qualitative research aims [30]; however, to increase the variations in our study population, the inclusions in the qualitative phase will be monitored to obtain an equal representation of different age and gender categories.

#### Recruitment and Enrollment

Before starting each part of the study, pilot phases will be performed to correct potential technical issues and ensure that the time for the completion of the survey and the interviews and focus groups do not exceed the expected duration (20 minutes for the survey and 60 and 90 minutes for individual interviews and focus groups, respectively).

The recruitment of participants will be based on electronic and paper flyers disseminated via social media, long COVID consultations, and long COVID patient associations in Luxembourg and neighboring countries (eg, France). Participation will also be proposed to participants from the Predi-COVID cohort study [2] who declared persisting symptoms at 1 year.

Flyers present information about the study and contain a direct link to the web-based survey so that interested persons can directly participate. The contact details of the study assistant at the LIH are also provided so that people can contact her directly if they are interested in participating in the qualitative part of the study.

People who expressed their interest in participating in the second part of the study, either after the survey completion or by contacting us directly, will be contacted by the study assistant. The study assistant will provide additional information regarding the second part of the project, and if the person confirms his or her interest in participating, an electronic ethical information notice and consent form will be sent to the participant. After signing the electronic consent form, the participant will be able to download a copy of the document. The study assistant will contact him or her to organize the individual interview. Electronic informed consent will be obtained before any data collection for the second part of the study.

Once the study assistant has received the notification that the participant has a valid signed consent, they will contact her or him to organize the web-based individual interview.

When all the individual interviews are completed, a panel comprising 7 to 8 people with long COVID and 1 to 2 HCPs will be constituted among the participants who expressed their willingness to participate in the focus group sessions.

When all individual interviews and focus groups are conducted, a restitution meeting will be organized. Everyone who has expressed an interest in this project will be invited.

#### Sample Size Calculation

For the quantitative part of the study, a formal size calculation was not feasible, given the exploratory nature of the study. The quantitative data from the survey will be exclusively used for descriptive purposes and to evaluate the different topics’ importance for the participants.

For the qualitative part, no gold standard for the “right” sample size exists and depends closely on the nature and aims of the study [31]. Qualitative studies do not aim to estimate magnitudes or generalize the results to a larger population but evaluate patterns in a data set. Participants will be sampled until data saturation is reached, which can be formalized as the point at which new data repeat what were already collected [32,33]. A recent systematic review showed that data saturation was achieved with a mean of 9 to 17 interviews but with high disparities between studies [34]. The ideal size for focus groups is estimated to be between 6 and 10 persons, particularly when including participants with high knowledge of the problem, which is the case for people with long COVID and HCPs [35].

To achieve data saturation, at least 15 people with long COVID and 5 HCPs involved in the management of patients with long COVID will participate in the individual semistructured interviews. However, these are estimations, and we will recruit participants until data saturation is reached.

A maximum of 7 to 8 people with long COVID and 1 to 2 HCPs will participate in 2 successive focus groups with an iterative co-design objective. Participants in the focus groups may be the same as those in the individual part, but this is not mandatory.

### Data Analysis

#### Phase 1: Quantitative Part

Quantitative data from the web-based survey will be analyzed using descriptive statistics methods. Continuous variables will be presented as means with SDs. Discrete numeric variables and ordinal variables will be presented as medians with IQRs. Nominal and categorical variables will be presented as proportions. Standard statistical methods will be followed.

#### Phase 2: Qualitative Part

Qualitative data from the semistructured interviews and focus groups will be audio recorded and transcribed verbatim. Transcripts will be checked for accuracy to ensure data quality and analyzed by using the thematic analysis approach [36]. This is the most commonly used method in qualitative research and allows one to identify, analyze, and report patterns within the data through the following five major steps:

1. Compiling: during this step, the recorded interviews are transcribed and organized.
2. Disassembling to allow for the creation of meaningful codes by identifying themes, concepts, or ideas. The coding process will be iterative and based on grounded theory [37].
3. Reassembling to further dive into the themes and creating subthemes. The coding process will be iterative until saturation.
4. Interpreting: thematic maps or code clusters will be created to analyze the relationship between the themes and thus answer the research question.
5. Concluding: conclusions are not generalizable to the population; they are merely an analytical generalization—a guide to assess how the findings can be transferred and applied. Indeed, qualitative research typically aims for in-depth understanding rather than statistical generalizability.

The MAXQDA software (VERBI Software GmbH) will be used for the transcription of the audio files, for coding and qualitative analysis.

Data analysis will be performed by the study investigators. In particular, coding will be performed by 2 researchers, including an experienced qualitative researcher.

On the basis of the data, the codes and the overall coding structure will be created by the researchers themselves, which is justified because the analysis will be based on grounded theory. Common themes and subthemes from the qualitative data will be identified using a thematic analysis. The first step will be performing coding from scratch. To facilitate coding, we will also create memos. We will clearly define each code that both researchers should approve. The 2 researchers will also have regular meetings to decide whether to merge or exclude codes (data reduction). The themes will be created by grouping similar codes. Subsequently, each theme will be revised to ensure a good definition that aligns with the required codes and to avoid redundancy or missing themes. In addition, we will define themes automatically, using nonsupervised machine learning algorithms (natural language processing using topic modelling R package [R Foundation for Statistical Computing]).

We will assess the intercoder reliability by double-blinded coding performed by 2 independent researchers. The Intercoder Agreement function from the MAXQDA software will be used to compare the coding of the 2 researchers involved in the coding process.

### Mixed Methods Integration and Analysis

The integration of the quantitative and qualitative data will be done twice. First, in the design of the qualitative phase of the study: the findings from the quantitative phase will be used to develop the semistructured interview guide that is to be used for the qualitative phase. In particular, the most important topics raised by the survey will be addressed first in the discussions.

Second, after the end of the qualitative phase, the results from the 2 phases will be integrated to triangulate the findings.

### Ethics Approval, Informed Consent, and Participation

This study was approved by the National Research Ethics Committee of Luxembourg (study number 202208/04) in August 2022. Interested persons can participate either in the 2 parts of the project or only in 1 part.

In the first part (quantitative web-based survey), each person who receives the link can participate. Detailed information on the survey is provided before acceding to the survey. Informed consent is not required, as this part is anonymous.

An extensive electronic information and informed consent form will be sent to people interested in participating in the qualitative part of the study. Electronically signed informed consent will be collected before any data collection for the second part of the study. REDCap will be used to manage the electronic consent forms.

Participants enrolled in the qualitative part may terminate their participation at any time by contacting the study assistant, and they will not be required to provide reasons for the decision. The assistant will complete the withdrawal form for our own records to ensure that the participant has declared whether LIH may continue to use the information that was already collected or if it should be destroyed (if applicable).

Participation in the individual interviews and the focus groups may tire some participants, particularly people with long COVID. To prevent this risk, regular pauses will be proposed during the interviews and focus groups, and the maximum duration of 60 and 90 minutes, respectively, will be strictly respected. The interviewer and the moderator will be the investigators trained in qualitative research methods and confidentiality.

There will be no financial incentive for participation in one or both parts of the study. Participants will be informed that they will not have any direct benefit but will contribute to a research effort that could make an impact not only in the care of patients with long COVID but also that of patients with other chronic diseases in the following years.

### Data Management—Confidentiality

The personal data of participants will be protected according to the *Regulation (EU) 2016/679 of the European Parliament and of the Council of 27 April 2016 on the protection of natural persons with regard to the processing of personal data and on the free movement of such data, and repealing Directive 95/46/EC (General Data Protection Regulation)*. The participants will receive detailed information on data privacy and protection in the information sheets from both parts of the study. In particular, they will be informed of how the data will be collected and stored, what security measures will be in place, and who will be authorized to access the data. They will also be asked to give their consent for the potential secondary use of their data for other research projects and will be informed that in case they agree, only their anonymized data will be shared with other researchers. Participants in the anonymous survey will be informed that their data will potentially be used for other research studies and shared with other researchers. The participants will be informed that they can contact the institution’s data protection officer if they have questions related to data privacy and protection. The contact details of the data protection officer are provided in the electronic information and consent form.

The survey will be anonymous and accessible by following a direct link to the web-based survey platforms (no application to download).

No account, log-in, or password will have to be created by the participants. There will be no link between the email addresses collected after the survey completion from people interested in the second part of the study and the survey answers.

The data collected from the qualitative part will be pseudonymized. Participants who express their interest in participating in the second part of the project will receive a unique link to access the electronic consent form. After the completion of the electronic consent form, a unique study number will be assigned to them.

Data collected during the individual interviews and focus groups will never be linked to a participant’s name or email address. A nickname will be used to refer to each participant during the exchanges. Interviews and focus groups will be recorded and transcribed using a transcription tool. All records will be destroyed directly after the transcription. The interviewer will be responsible for audio transcription and destruction.

The nominative data collected via the consent form will be stored on LIH internal servers, separated from the pseudonymized data from the interviews and focus group notes. All data stored on the LIH servers will be encrypted using the Transport Layer Security (TLS) encryption protocol. Access to the data will be restricted by user accounts and will be provided by the principal investigator of the study to a limited number of people. Access logs will be implemented to control data accesses and ensure high data security.

The contact data (email address and identification data) of the participants will be deleted 2 years after the individual interview. The pseudonymized data will be anonymized after 2 years by deleting the correspondence table and deleted after 10 years according to the LIH standard procedure for archiving.

LIH internal procedures will be followed to ensure data destruction after the retention period ends.

## Results

The web-based survey was launched in October 2022. The individual interviews and focus groups will start after the end of the survey in March 2023. The end of data collection is expected in September 2023, and the results will be published at the end of 2023 or in 2024.

## Discussion

This paper presents a protocol for a mixed methods study that aims to codevelop a digital health solution based on vocal biomarkers to monitor long COVID symptoms and conditions with its potential end users, namely, people with long COVID and HCPs in charge of patients with long COVID.

### Expected Findings

Long COVID is a complex disease with a high impact on the daily life and quality of life of affected people [38,39]. Patients often feel abandoned by the health care system and demand for support and new tools to track their symptoms and relapses. This could be helpful to identify the potential causal factors of symptom exacerbation and relapses to improve pacing and prevention. In particular, postexertional symptom exacerbation is a well-known problem in long COVID and can be defined as the worsening of symptoms following a physical or mental activity [40]. Postexertional symptom exacerbation needs to be carefully monitored, for example, to ensure a safe rehabilitation program or to personalize the daily activities to prevent symptom degradation.

HCPs also recognize that there is a need for more integrated health care structures to manage patients with long COVID. Additional tools to ensure an efficient and safe rehabilitation would also be helpful for both people with long COVID and HCPs, as some patients with long COVID experience postexertional malaise [40] and may need a tailored rehabilitation program. Moreover, with new therapies and drugs currently in development for long COVID treatment, this tool might also be used as a companion tool to monitor the health status of people with long COVID during clinical trials.

The use of vocal biomarkers to monitor long COVID symptoms seems of high interest because voice samples are noninvasively collected and are easy to collect compared with the completion of tedious self-reported questionnaires. They could also objectify a subjective condition such as fatigue or mental health and facilitate the communication between patients and HCPs [10,41]. Moreover, audio recordings may be more adapted and accurate than traditional paper questionnaires for people with neuropsychological disorders or other patients who are chronically ill with reduced functioning [42,43].

Vocal biomarkers of several symptoms related to COVID-19 and long COVID have been identified previously and need to be integrated into a digital health solution before being used in daily life or clinical practice. We recently proposed an optimal pipeline and recommendations to achieve the successful implementation of vocal biomarkers in practice, with their potential benefits and limitations in the context of long COVID [41]. The identification of the problem and the symptoms to be monitored and the involvement of the end users as soon as possible in the development process are key points. The protocol of the study described in this paper is based on these recommendations.

The findings of this study will have several potential implications. First, we will take advantage of combining quantitative and qualitative data in a mixed methods design to describe the needs of people with long COVID in depth and understand how the use of vocal biomarkers in a voice-based digital health solution could solve their problems. The visualization of the results in the digital health solution will also be discussed and tailored to best fit its end users. The tool will be developed with an interoperability objective to ensure its future dissemination to a maximum number of people.

Second, the findings could potentially be transferred to other diseases, and this is of the highest importance for the future of vocal biomarkers and voice-based health technologies to understand the extent of transferability.

Finally, the concrete output of the study will be a voice-based digital health solution that will provide support to people with long COVID in their daily lives and improve their quality of life.

### Strengths and Limitations

One of the strengths of this study is that patients with long COVID and HCPs in charge of patients with long COVID have been involved in the study design and will be involved in the entire study course.

The use of a mixed methods design, using quantitative and qualitative approaches, will provide an extensive description of the future user’s expectancies, barriers, and leverages regarding the use of vocal biomarkers in a digital health solution and will provide the specifications of a digital health solution optimally designed with and for its end users.

The results will reveal the transferability potential of the use of vocal biomarkers for other diseases and contribute to the expansion of this recent technology.

This study has some limitations. First, the fully web-based format of the study may limit the participation of some population categories (such as people with low eHealth literacy) but by contrast, may also facilitate the participation of people who are geographically distant or experience more impacting symptoms.

Another potential limitation is that even if the chosen sample size should ensure achieving data saturation, it may probably not represent all different subpopulations with long COVID. However, this is not the objective of this mixed methods study. The opinions collected should be varied enough to ensure the development of a digital solution that provides help and support to all patients with long COVID.

Finally, mixing people with long COVID and HCPs in the focus groups may induce conformity, dominance, and shyness biases. However, people with long COVID are highly involved in the management of their disease and generally interact easily with HCPs. The moderator’s role will also be crucial in minimizing the risks of bias during focus groups by communicating from the start the objectives of the discussions and by ensuring that each participant receives the same amount of speaking time. The cultural bias will also be reduced by the inclusion of participants from different countries.

### Conclusions

This mixed methods study will identify the needs of people affected by long COVID in their daily lives and describe the main symptoms or problems that would need to be monitored and improved. We will determine how the use of vocal biomarkers in a digital health solution could meet these needs and further describe the end user’s expectations and barriers regarding the use of this new digital technology to monitor COVID-19–related symptoms. Finally, our study will ensure the development of a digital health solution that will match the actual needs of people with long COVID by involving them in the design process from the beginning. The study will also provide important insights into the transferability of the results to other diseases and contribute to the deployment of vocal biomarkers in general.

### Publication Plan

The study results will be disseminated at the end of 2023 in a scientific article submitted to an international peer-reviewed journal, to long COVID patient associations, and through social media communications to the lay audience.

## Appendix

Multimedia Appendix 1 Detailed questionnaire for people with long COVID.

Multimedia Appendix 2 Detailed health care professional questionnaire.

## Abbreviations

APEASE: Affordability, Practicability, Effectiveness, Acceptability, Side Effects, and Equity
HCP: health care professional
LIH: Luxembourg Institute of Health
REDCap: Research Electronic Data Capture
TLS: Transport Layer Security
uMARS: user version of the Mobile Application Rating Scale

## Data Availability

The data sets generated during this study are available from the corresponding author upon reasonable request.
