# Supplementary material for: Co-Design of a Voice-Based Digital Health Solution to Monitor Persisting Symptoms Related to COVID-19 (UpcomingVoice Study): Protocol for a Mixed Methods Study
Source: JMIR Res Protoc. 2023 Jun 19;12:e46103. doi: 10.2196/46103 (PMC10337302; doi:10.2196/46103)
Supplement: Multimedia Appendix 1 [file resprot_v12i1e46103_app1.docx]

**Table S1: People with long COVID survey (in French)**

#

|  | **QUESTION** | **RÉPONSE** |
| --- | --- | --- |
| Q1 | De quel sexe êtes-vous ? | ☐ Masculin  ☐ Féminin  ☐ Autre |
| Q2 | Quel est votre âge ? | *☐ ☐* |
| Q3 | Quel est le plus haut diplôme ou niveau d’études que vous ayez atteint ? | ☐ Aucun diplôme  ☐ Certificat d’éducation générale, certificat d’éducation primaire, certificat de fin d’études  ☐ Certificat de compétence professionnelle, certificat de formation professionnelle  ☐ Baccalauréat ou diplôme équivalent  ☐ Bac +2 ou +3  ☐ Bac +4  ☐ Bac +5 ou plus  ☐ Autre :_ |

#

| Q4 | Avez-vous déjà été testé(e) positif(ve) à la Covid-19 (Sars-Cov-2) ?* | ☐ Oui  ☐ Non  ☐ Je ne sais pas |
| --- | --- | --- |
| Q5 | **Si oui*: Avez-vous été hospitalisé(e) pour la Covid-19 ? | ☐ Oui  ☐ Non |
| *Q6* | *Si oui* : avez-vous été hospitalisé en... | ☐ Service général, **pendant** : *☐ ☐* jour/semaine/mois  ☐ Soins intensifs, **pendant** : *☐ ☐* jour/semaine/mois |
| Q7 | **Si oui*: Au moment où vous avez été testé(e), avez-vous présenté des symptômes ? | ☐ Oui, symptômes sévères  ☐ Oui, symptômes légers  ☐ Non  ☐ Je ne sais pas |
| Q8 | Avez-vous présenté des symptômes **persistants** (pendant au moins 3 semaines ou plus et plus de 2 mois après l’infection aigüe) ou de nouveaux symptômes qui n’étaient pas présents au cours des 15 premiers jours ? | ☐ Oui*  ☐ Non  ☐ Je ne sais pas |
| Q9 | **Si oui*:comment qualifieriez-vous l’intensité de vos symptômes ? | ☐ Symptômes sévères  ☐ Symptômes légers  ☐ Je ne sais pas |
| Q10 | Est-ce qu’un médecin vous a dit que vous aviez un Covid long ? | ☐ Oui  ☐ Non, mais mes symptômes sont très évocateurs  ☐ Je ne sais pas |
| Q11 | Avez-vous bénéficié d’une consultation chez un médecin spécialiste du Covid Long ou dans un réseau de consultation COVID Long ? | ☐ Oui  ☐ Non  ☐ Je ne sais pas |
| Q12 | Dans quelle mesure a-t-il été difficile ou facile d’accéder aux services de santé dont vous avez besoin depuis l’apparition de vos symptômes ? | ☐ Très difficile  ☐ Difficile  ☐ Moyennement difficile  ☐ Facile  ☐ Très facile  ☐ Je ne sais pas |
| Q13 | Etes-vous vacciné(e) contre la Covid-19 ? | ☐ Oui  ☐ Non  ☐ Je ne sais pas |
| Q14 | Avez-vous pu reprendre vos activités (professionnelles, loisirs etc..) au même rythme qu’avant l’apparition des symptômes ? | ☐ Oui  ☐ Non  ☐ Je ne sais pas |
| Q15 | Pensez-vous qu’une application digitale pourrait vous aider à gérer votre état de santé sur le long terme? | ☐ Oui  ☐ Non  ☐ Je ne sais pas |

| Q16 | Aviez-vous déjà entendu parler de biomarqueurs vocaux avant cette étude ? | ☐ Oui  ☐ Non  ☐ Ne sais pas |
| --- | --- | --- |
| Q17 | Dans quelle mesure acceptez-vous l’idée que des symptômes puissent être mesurés dans la voix grâce aux biomarqueurs vocaux ? | ☐ Tout à fait convaincu  ☐ Convaincu  ☐ Moyennement convaincu  ☐ Pas du tout convaincu |
| Q18 | Seriez-vous prêt(e) à utiliser une solution de suivi de votre santé basée sur la voix ? | ☐ Oui  ☐ Non  ☐ Je ne sais pas |
| Q19 | Si non, pourquoi ? | ☐ Je n’aime pas enregistrer ma voix  ☐ J’ai peur du risque de fuite de données/du non-respect de ma vie privée  ☐ Cela ne me semble pas une technologie fiable  ☐ Je ne suis pas à l’aise avec la technologie en général  ☐ Autre : ….  ☐ Je ne sais pas |
| Q20 | Dans quelle mesure seriez-vous intéressé(e) par une application smartphone basée sur les biomarqueurs vocaux pour suivre votre santé ? | ☐ Très intéressé(e)  ☐ Intéressé(e)  ☐ Moyennement intéressé(e)  ☐ Faiblement intéressé(e)  ☐ Pas du tout intéressé(e) |
| Q21 | Dans quelle mesure pensez-vous qu’une telle solution digitale pourrait être utile pour les personnes ayant un Covid Long ? | ☐ Très utile  ☐ Utile  ☐ Moyennement utile  ☐ Peu utile  ☐ Pas utile du tout |
| Q22 | Quels sont selon vous les freins à l’utilisation d’une telle application ? | ☐ Intensité des symptômes du Covid long  ☐ Age des utilisateurs  ☐ Craintes liées à la protection des données et de la vie privée  ☐ Craintes d’une mauvaise interprétation des résultats  ☐ Trop d’applications de santé  ☐ Technologie trop récente  ☐ Coût  ☐ Autre ……  ☐ Je ne sais pas |
| Q23 | Quels sont les avantages potentiels d’une telle application selon vous ? | ☐ Suivre l’évolution de mes symptômes de manière régulière  ☐ Limiter mes déplacements pour des rdv médicaux  ☐ Evaluer l’efficacité d’un programme de rééducation  ☐ Avoir une mesure objective de mes symptômes sans remplir de long questionnaire  ☐ Autre ….  ☐ Je ne sais pas |
| Q24 | Quels sont les symptômes (directement ou indirectement liés au Covid Long) qui vous semblent les plus pénibles ou les plus importants à être suivis à distance ? (choix multiples) | ☐ Essoufflement  ☐ Fatigue  ☐ Présence - absence de symptômes  ☐ Perte goût ou odorat  ☐ Stress / anxiété  ☐ Symptômes gastro intestinaux  ☐ Symptômes neurologiques  ☐ Autre? …..  ☐ Je ne sais pas |
| Q25 | La solution devrait-elle être axée sur le suivi d’un seul symptôme (le plus invalidant) ou d’un ensemble de plusieurs symptômes ? | ☐ Un seul symptôme  ☐ Plusieurs symptômes  ☐ Je ne sais pas |
| Q26 | Choisissez les 3 caractéristiques de ce type d’application les plus importantes à vos yeux ? | ☐ Facilité d’utilisation  ☐ Sécurité des données/protection de ma vie privée  ☐ Visualisation des résultats d’analyse des biomarqueurs vocaux  ☐ Fiabilité  ☐ Esthétique  ☐ Aspect ludique  ☐ Rapidité d’analyse |
| Q27 | Dans quelles situations cette application vous paraît-elle utile ? | ☐ Suivre l’évolution d’un symptôme en particulier au cours d’un programme de rééducation  ☐ Suivre l’évolution de plusieurs symptômes simultanément  ☐ Obtenir un “diagnostic” de Covid Long  ☐ Autre …. |
| Q28 | Une application de santé intégrant des biomarqueurs vocaux pour suivre les symptômes liés au Covid Long devrait-elle être remboursée ? | ☐ Oui  ☐ Non  ☐ Je ne sais pas |
| Q29 | Si oui pourquoi ? (choix multiples) | ☐ Amélioration de la qualité de vie des personnes concernées  ☐ Prise en charge plus précoce  ☐ Réduction des coûts de prise en charge  ☐ Meilleure adhérence aux traitements ou programmes de rééducation  ☐ Autre : …..  ☐ Ne sais pas |
| Q30 | Si non seriez-vous prêt(e) à payer pour ce type de technologie si votre médecin ou une autre personne ayant un Covid Long vous la conseillait ? | ☐ Oui  ☐ Non  ☐ Je ne sais pas |
| Q31 | Si oui combien ? | ☐ Moins de 5 euros par mois  ☐ Entre 5 et 10 euros par mois  ☐ Entre 10 et 20 euros par mois  ☐ Plus de 10 euros par mois (combien?)  ☐ Autre : …..  ☐ Ne sais pas |
| Q32 | Quelles options de personnalisation vous semblent nécessaires ? | ☐ Taille texte  ☐ Sons  ☐ Activer ou non notifications  ☐ Choisir un avatar  ☐ Insérer photo de profil  ☐ Type d’affichage des résultats  ☐ Choix des informations à afficher  ☐ Autre…  ☐ Je ne souhaiterais pas personnaliser l’application |
| Q33 | Aimeriez-vous avoir la possibilité d’interagir avec d’autres utilisateurs ? | ☐ Oui, par le biais d’un forum de discussion  ☐ Oui, par le biais d’une messagerie sécurisée  ☐ Non  ☐ Je ne sais pas |
| Q34 | Aimeriez-vous avoir la possibilité de donner votre avis sur l'application en elle-même à ses concepteurs ? | ☐ Oui  ☐ Non  ☐ Je ne sais pas |
| Q35 | Attachez-vous de l’importance au côté ludique pour une application de ce type ? | ☐ Oui  ☐ Non  ☐ Je ne sais pas |
| Q36 | Pensez-vous qu'une application de ce type devrait proposer des informations ? | ☐ Oui  ☐ Non  ☐ Je ne sais pas |
| Q37 | Quels types d’informations vous semblent intéressantes à intégrer? | ☐ Explications sur la réalisation des enregistrements vocaux  ☐ Informations sur les biomarqueurs vocaux  ☐ Informations médicales sur le Covid Long  ☐ Module proposant des exercices de rééducation  ☐ Conseils personnalisés en fonction des résultats  ☐ Autre…  ☐ Je ne sais pas |
| Q38 | Quels types de conseils personnalisés (en fonction des symptômes que vous présentez) souhaiteriez-vous obtenir ? | ☐ Conseils alimentaires  ☐ Recommandations d’activité physique  ☐ Amélioration du sommeil  ☐ Amélioration du bien-être psychologique (exercices de méditation par exemple)  ☐ Autre ….  ☐ Aucun  ☐ Je ne sais pas |
| Q39 | Quelle fréquence vous semble acceptable pour la réalisation des enregistrements ? | ☐ Une fois par semaine  ☐ Deux fois par semaine  ☐ Trois fois par semaine  ☐ Tous les jours  ☐ A chaque fois que j’en ressens le besoin  ☐ Je ne sais pas |
| Q40 | Quels types d’enregistrements accepteriez-vous de réaliser régulièrement ? | ☐ Dire une voyelle (par exemple le son Aaaa) le plus longtemps possible  ☐ Compter de 1 à 20  ☐ Respirer profondément plusieurs fois  ☐ Tousser  ☐ Lire un court texte prédéfini  ☐ Répondre à une question simple  ☐ Enregistrement libre pour dire comment vous vous sentez  ☐ Enregistrement libre sur n’importe quel sujet de votre choix  ☐ Je ne sais pas |
| Q41 | Accepteriez-vous d’effectuer plusieurs enregistrements consécutifs lors d’une utilisation de l’application ? | ☐ Oui  ☐ Non  ☐ Je ne sais pas |
| Q42 | Quel temps maximum seriez-vous prêts à utiliser l’application chaque semaine ? | ☐ Moins d’1 heure  ☐ Entre 1 heure et 2 heures  ☐ Entre 2 et 4 heures  ☐ Plus de 4 heures  ☐ Je ne sais pas |
| Q43 | Souhaiteriez-vous pouvoir partager vos résultats avec un professionnel de santé de votre choix ou un membre de votre famille par exemple ? | ☐ Oui  ☐ Non  ☐ Je ne sais pas |
| Q44 | Si oui de quelle manière ? | ☐ Automatiquement via l'application (envoi d’un mail à un professionnel de santé ou autre personne désignée)  ☐ En téléchargeant un rapport PDF et en le partageant soi-même avec son professionnel de santé ou autre personne de son choix)  ☐ Autre : …  ☐ Je ne sais pas |
| Q45 | Aimeriez-vous pouvoir synchroniser l’application avec les données d’autres applications de santé que vous utilisez (app Santé, Google Fit, Apple Health, Samsung Health etc…) ou avec des objets connectés (montre, balance etc..) ? | ☐ Oui  ☐ Non  ☐ Je ne sais pas |
| Q46 | Dans quelle situation penseriez-vous réaliser les enregistrements vocaux requis par la solution digitale? | ☐ Uniquement si je suis certain(e) d’être seul(e)  ☐ Seul(e) ou en présence de ma famille(à la maison)  ☐ Uniquement en présence d’un professionnel de santé  ☐ N’importe où (travail, maison, à l’extérieur)  ☐ Je ne sais pas |
| Q47 | Pensez-vous que la solution digitale devrait être basée uniquement sur des enregistrements de voix ou également sur d’autres données? | ☐ Uniquement des enregistrements de voix, pour la simplicité d’utilisation  ☐ Des enregistrements de voix complétés par des questionnaires réguliers  ☐ Autre : …  ☐ Je ne sais pas |
| Q48 | Imaginons que l’utilisateur de l’application ait un niveau élevé du biomarqueur vocal de santé mentale (symptômes dépressifs liés au Covid Longs) détecté lors de l’analyse de sa voix. Pensez-vous qu’un système d’alerte doit être intégré à l’application ? | ☐ Oui  ☐ Non  ☐ Je ne sais pas |
| Q49 | Si oui sous quelle forme ? | ☐ Notification  ☐ Mail  ☐ SMS  ☐ Autre …  ☐ Je ne sais pas |
| Q50 | Si oui, qui devrait recevoir ces alertes ? | ☐ Les utilisateurs eux-même  ☐ Une personne désignée par l’utilisateur lui-même  ☐ Le médecin spécialiste en charge du patient (si applicable)  ☐ L’équipe de soin (infirmiers, kinésithérapeutes..) en charge du patient (si applicable)  ☐ Autre …  ☐ Je ne sais pas |
| Q51 | Souhaiteriez-vous avoir la possibilité d’afficher un calendrier avec les dates de vos enregistrements et les résultats obtenus? | ☐ Oui  ☐ Non  ☐ Ne sais pas |
| Q52 | Souhaiteriez-vous avoir la possibilité d’intégrer un calendrier de votre programme de rééducation (si vous en avez un)l? | ☐ Oui  ☐ Non  ☐ Ne sais pas |
| Q53 | L’application devrait-elle proposer un module de rééducation ? | ☐ Oui  ☐ Non  ☐ Ne sais pas |
| Q54 | Si oui quels types d’exercices de rééducation pourraient être intégrés ? | ☐ Exercices de stimulation cérébrale  ☐ Exercices de renforcement musculaire  ☐ Exercices de respiration  ☐ Exercices pour récupération goût et odorat  ☐ Autre : …  ☐ Ne sais pas |
| Q55 | Comment devraient être proposés les programmes de rééducation ? | ☐ Tous les modules devraient être présents et l’utilisateur fera le choix de ses exercices en fonction de ses besoins  ☐ L’application devrait proposer un programme d’exercices en fonction des symptômes détectés  ☐ Autre : …  ☐ Ne sais pas |
| Q56 | Quelles autres fonctionnalités devraient-elles être intégrées à une application de ce type?: | ☐ Système de rappels pour la réalisation des enregistrements  ☐ Système d’encouragement (badges)  ☐ Forum de discussion  ☐ Messagerie (avec son professionnel de santé)  ☐ Chat entre personnes avec un Covid Long  ☐ Autre : …  ☐ Je ne sais pas |
| Q57 | Sous quelle forme souhaiteriez-vous obtenir les résultats des biomarqueurs vocaux de vos différents symptômes? | ☐ Scores représentés sous forme de couleurs correspondant à de grandes catégories (ex : vert pour un résultat normal, orange pour un résultat intermédiaire et rouge pour un résultat anormal)  ☐ Scores, sous forme de chiffres (par ex : une échelle de 0 à 10)  ☐ Scores de 0 à 10 représentés sous forme graphique  ☐ Autre : …  ☐ Je ne sais pas |
