# Supplementary material for: Co-Design of a Voice-Based Digital Health Solution to Monitor Persisting Symptoms Related to COVID-19 (UpcomingVoice Study): Protocol for a Mixed Methods Study
Source: JMIR Res Protoc. 2023 Jun 19;12:e46103. doi: 10.2196/46103 (PMC10337302; doi:10.2196/46103)
Supplement: Multimedia Appendix 2 [file resprot_v12i1e46103_app2.docx]

**Table S2: Healthcare professional survey (in French)**

|  | **QUESTION** | **RÉPONSE** |
| --- | --- | --- |
| HCP-Q1 | De quel sexe êtes-vous ? | ☐ Masculin  ☐ Féminin  ☐ Autre |
| HCP-Q2 | Quel est votre âge ? | *☐ ☐* |
| HCP-Q3 | Quel est le plus haut diplôme ou niveau d’études que vous ayez atteint ? | ☐ Certificat d’éducation générale, certificat d’éducation primaire, certificat de fin d’études  ☐ Certificat de compétence professionnelle, certificat de formation professionnelle  ☐ Baccalauréat ou diplôme équivalent  ☐ Bac +2 ou +3  ☐ Bac +4  ☐ Bac +5 ou plus  ☐ Autre :_ |

#

| HCP-Q4 | Aviez-vous déjà entendu parler de biomarqueurs vocaux avant cette étude ? | ☐ Oui  ☐ Non  ☐ Je ne sais pas |
| --- | --- | --- |
| HCP-Q5 | Dans quelle mesure acceptez-vous l’idée que des symptômes puissent être mesurés dans la voix grâce aux biomarqueurs vocaux ? | ☐ Tout à fait convaincu  ☐ Convaincu  ☐ Moyennement convaincu  ☐ Pas du tout convaincu  ☐ Je ne sais pas |
| HCP-Q6 | Dans quelle mesure pensez-vous qu’une application mobile pourrait être utile pour les personnes ayant un Covid Long et non prises en charge par un réseau spécialisé de prise en charge Covid Long ? | ☐ Très utile  ☐ Utile  ☐ Moyennement utile  ☐ Peu utile  ☐ Pas utile du tout  ☐ Je ne sais pas |
| HCP-Q7 | Dans quelle mesure pensez-vous qu’une application mobile pourrait être utile pour les personnes ayant un Covid Long et déjà prises en charge par un réseau spécialisé de prise en charge Covid Long ? | ☐ Très utile  ☐ Utile  ☐ Moyennement utile  ☐ Peu utile  ☐ Pas utile du tout  ☐ Je ne sais pas |
|  | Cette application mobile, grâce à laquelle les personnes ayant un Covid Long ou qui pensent avoir un Covid Long pourraient enregistrer leur voix avec leur smartphone, l’application pourrait alors mesurer les niveaux de biomarqueurs vocaux de différents symptômes du Covid Long et en informer la personne. Ceci permettrait à la personne de suivre l’évolution d’un symptôme pendant sa rééducation ou de dépister un Covid Long. |  |
| HCP-Q8 | Conseilleriez-vous à vos patients d’utiliser une application de ce type ? | ☐ Oui  ☐ Non  ☐ Je ne sais pas |
|  | Si oui, pourquoi ? | …….. |
|  | Si non, pourquoi ? | ……. |
| HCP-Q9 | Quels sont selon vous les freins à l’utilisation d’une telle application ? | ☐ Intensité des symptômes du Covid Lond  ☐ Age des utilisateurs  ☐ Craintes liées à la protection des données/vie privée  ☐ Craintes d’une mauvaise interprétation des résultats  ☐ Trop d’application de santé  ☐ Technologie trop récente (BV)  ☐ Coût  ☐ Autre ….  ☐ Je ne sais pas |
| HCP-Q10 | Quels sont les avantages d’une telle solution selon vous pour les patients ? | ☐ Suivre l’évolution de leurs symptômes de manière régulière (amélioration ou dégradation)  ☐ Diagnostiquer un Covid Long chez des personnes en errance médicale  ☐ Evaluer l’efficacité d’un programme de rééducation  ☐ Limiter leurs déplacements pour des rdv médicaux  ☐ Avoir une mesure objective des symptômes sans remplir de long questionnaire  ☐ Etre un outil d’accompagnement  ☐ Améliorer l’adhérence aux traitements ou aux programmes de rééducation  ☐ Alerter les personnes en cas d'aggravation de leur état de santé  ☐ Autre ….  ☐ Je ne sais pas |
| HCP-Q11 | Quels sont les symptômes (directement ou indirectement liés au Covid Long) qui vous semblent les plus pénibles ou les plus importants à être suivis à distance ? (choix multiples) | ☐ Cardiorespiratoires (dyspnée, hypertension..)  ☐ Fatigue  ☐ Présence - absence de symptômes  ☐ Perte goût ou odorat  ☐ Stress / anxiété  ☐ Symptômes gastro intestinaux  ☐ Symptomes neurologiques  ☐ Autre …..  ☐ Je ne sais pas |
| HCP-Q12 | Pour un patient donné, la solution devrait-elle être axée sur le suivi d’un seul symptôme (le plus impactant sur la qualité de vie) ou d’un ensemble de plusieurs symptômes ? | ☐ Un seul symptôme  ☐ Plusieurs symptômes  ☐ Je ne sais pas |
| HCP-Q13 | Choisissez les 3 caractéristiques de ce type d’application les plus importantes à vos yeux ? | ☐ Facilité d’utilisation  ☐ Sécurité des données  ☐ Visualisation des résultats d’analyse des biomarqueurs vocaux  ☐ Fiabilité  ☐ Esthétique  ☐ Aspect ludique  ☐ Rapidité d’analyse |
| HCP-Q14 | Dans quelles situations cette application vous paraît-elle utile ? | ☐ Suivre l’évolution d’un symptôme en particulier au cours d’un programme de rééducation  ☐ Suivre l’évolution de plusieurs symptômes simultanément  ☐ Etablir un “diagnostic” de Covid Long  ☐ Autre …. |
| HCP-Q15 | Une application de santé intégrant des biomarqueurs vocaux pour suivre les symptômes liés au Covid Long devrait-elle être remboursée ? | ☐ Oui  ☐ Non  ☐ Ne sais pas |
| HCP-Q16 | Si oui pourquoi ? (choix multiples) | ☐ Amélioration de la qualité de vie des personnes concernées  ☐ Prise en charge plus précoce  ☐ Réduction des coûts de prise en charge  ☐ Meilleure adhérence aux traitements ou programmes de rééducation  ☐ Autre : …..  ☐ Ne sais pas |
| HCP-Q17 | Pensez-vous que les patients seraient prêts à payer pour une telle application si elle n'était pas remboursée ? | ☐ Oui  ☐ Non  ☐ Ne sais pas |
| HCP-Q18 | Si oui combien ? | ☐ Moins de 5 euros par mois  ☐ Entre 5 et 10 euros par mois  ☐ Entre 10 et 20 euros par mois  ☐ Plus de 10 euros par mois (combien?)  ☐ Autre : …..  ☐ Ne sais pas |
| HCP-Q19 | Pensez-vous qu'une application de ce type devrait proposer des informations? | ☐ Oui  ☐ Non  ☐ Je ne sais pas |
| HCP-Q20 | Quels types d’informations vous semblent intéressantes à intégrer? | ☐ Explications sur la réalisation des enregistrements vocaux  ☐ Informations sur les biomarqueurs vocaux  ☐ Informations médicales sur le Covid Long  ☐ Module proposant des exercices de rééducation  ☐ Conseils personnalisés en fonction des résultats  ☐ Autre…  ☐ Je ne sais pas |
| HCP-Q21 | Quels types de conseils personnalisés souhaiteriez-vous obtenir ? | ☐ Conseils alimentaires  ☐ Recommandations d’activité physique  ☐ Amélioration du sommeil  ☐ Amélioration du bien-être psychologique (exercices de méditation par exemple)  ☐ Autre ….  ☐ Aucun  ☐ Je ne sais pas |
| HCP-Q22 | Quels types d’enregistrements vous semblent réalisables par des personnes ayant un Covid Long? | ☐ Dire une voyelle (par exemple le son Aaaa) le plus longtemps possible  ☐ Compter de 1 à 20  ☐ Respirer profondément plusieurs fois  ☐ Tousser  ☐ Lire un court texte prédéfini  ☐ Répondre à une question simple  ☐ Enregistrement libre pour dire comment ils se sentent  ☐ Enregistrement libre sur n’importe quel sujet de leur choix  ☐ Ne sais pas |
| HCP-Q23 | Quelle fréquence vous semble la plus appropriée pour la réalisation des enregistrements? | ☐ Une fois par semaine  ☐ Deux fois par semaine  ☐ Trois fois par semaine  ☐ Tous les jours  ☐ A chaque fois que le patient en ressent le besoin  ☐ Je ne sais pas |
| HCP-Q24 | Pensez-vous que la solution digitale devrait être basée uniquement sur des enregistrements de voix ou également sur d’autres données? | ☐ Uniquement des enregistrements de voix, pour la simplicité d’utilisation  ☐ Des enregistrements de voix complétés par des questionnaires réguliers  ☐ Autre : …  ☐ Je ne sais pas |
| HCP-Q25 | Pensez-vous qu’il est important que l’application donne la possibilité de partager les résultats avec un professionnel de santé au choix ? | ☐ Oui  ☐ Non  ☐ Je ne sais pas |
| HCP-Q26 | Pensez-vous que la solution développée devrait être couplée à un système d’alerte en cas de résultats anormaux ? | ☐ Oui  ☐ Non  ☐ Je ne sais pas |
| HCP-Q27 | Imaginons que l’utilisateur de la solution digitale ait un niveau élevé du biomarqueur vocal de santé mentale (symptômes dépressifs) détecté lors de l’analyse de sa voix. Pensez-vous qu’un système d’alerte soit intégré à la solution digitale de santé ? | ☐ Oui  ☐ Non  ☐ Je ne sais pas |
| HCP-Q28 | Si oui sous quelle forme? | ☐ Notification  ☐ Mail  ☐ SMS  ☐ Autre …  ☐ Je ne sais pas |
| HCP-Q29 | Si oui, qui devrait recevoir ces alertes? | ☐ Les patients eux-même  ☐ Une personne désignée par l’utilisateur lui-même  ☐ Le médecin spécialiste en charge du patient (si applicable)  ☐ L’équipe de soin (infirmiers, kinésithérapeutes..) en charge du patient (si applicable)  ☐ Autre …  ☐ Je ne sais pas |
|  |  |  |
| HCP-Q30 | Pensez-vous qu’il soit utile de pouvoir intégrer le programme de rééducation éventuel du patient ? | ☐ Oui  ☐ Non  ☐ Ne sais pas |
| HCP-Q31 | L’application devrait-elle proposer un module de rééducation ? | ☐ Oui  ☐ Non  ☐ Ne sais pas |
| HCP-Q32 | Si oui quels types d’exercices de rééducation pourraient être intégrés ? | ☐ Exercices de stimulation cérébrale (type NeuronationsMed)  ☐ Exercices de renforcement musculaire  ☐ Exercices de respiration  ☐ Exercices pour récupération goût et odorat  ☐ Autre : …  ☐ Ne sais pas |
| HCP-Q33 | Comment devraient être proposés les programmes de rééducation ? | ☐ Tous les modules devraient être présents et l’utilisateur fera le choix de ses exercices en fonction de ses besoins  ☐ L’application devrait proposer un programme d’exercices en fonction des symptômes détectés  ☐ Autre : …  ☐ Ne sais pas |
| HCP-Q34 | Quelles autres fonctionnalités devraient-elles être intégrées à une application de ce type ? | ☐ Système de rappels pour la réalisation des enregistrements  ☐ Système d’encouragement (badges)  ☐ Forum de discussion  ☐ Messagerie  ☐ Autre …  ☐ Aucun  ☐ Je ne sais pas |
| HCP-Q35 | Quelle forme vous semble la plus appropriée pour représenter les résultats des biomarqueurs vocaux des différents symptômes? | ☐ Scores représentés sous forme de couleurs correspondant à de grandes catégories (ex : vert pour un résultat normal, orange pour un résultat intermédiaire et rouge pour un résultat anormal)  ☐ Scores, sous forme de chiffres (par ex : une échelle de 0 à 10)  ☐ Scores de 0 à 10 représentés sous forme graphique  ☐ Autre : …  ☐ Je ne sais pas |
